# A prefrontal cortex alpha/delta switch controls the transition from positive to negative affective states

**DOI:** 10.1007/s44192-023-00044-3

**Published:** 2023-10-10

**Authors:** Jeffrey S. Burgdorf, Joseph R. Moskal

**Affiliations:** 1 Falk Center for Molecular Therapeutics, Department of Biomedical Engineering, McCormick School of Engineering, Northwestern University, Evanston, IL 60201 USA; 2 Present Address: Gate Neurosciences Inc., Carmel, IN 46032 USA

**Keywords:** Positive affect, ultrasonic vocalizations, quantiative EEG, NMDA receptors, AMPA receptors

## Abstract

Positive and negative emotional states in rats can be studied by investigating ultrasonic vocalizations (USVs). Positive affect in rats is indexed by 50 kHz hedonic USVs, and negative affect is indexed by 22 kHz aversive calls. We examined the relationship of emotional states in rats using medial prefrontal cortex (MPFC) quantitative electroencephalograms (qEEG) and found that hedonic USVs were associated with active wake qEEG (high alpha/low delta power), and aversive USVs occurred with groggy wake qEEG (low alpha/high delta). Further, alpha frequency electrical stimulation of the MPFC induces hedonic calls and reward-seeking behavior, whereas delta frequency stimulation produces aversive calls and avoidance behavior. The brain region responsible for generating motor output for USVs, the periaqueductal gray (PAG), shows a motor-evoked potential that is temporally locked to the alpha (hedonic) and delta (aversive) motor-evoked potential. Closed-loop alpha frequency electrical stimulation could prevent delta qEEG and aversive USVs. At the neuronal circuit level, the alpha rhythm was associated with synaptic long-term potentiation (LTP) in the cortex, whereas the delta rhythm was associated with synaptic depotentiation (LTD) in the cortex. At the pharmacological level, NMDAR and growth factor modulation regulated these forms of neuroplasticity. At the single neuron level, excitatory neurons show increased activity in response to alpha frequencies and decreased activity during delta frequencies. In humans, the feeling of joy increased alpha and decreased delta power in frontal scalp qEEG, and the opposite response was seen for sadness. Thus, the synchronization of alpha/delta oscillations through the neuronal circuit responsible for emotional expression coordinates emotional behavior, and the switch between active wake/positive affect and groggy wake/negative affect is under the control of an LTP- LTD synaptic plasticity mechanism.

## Introduction

Positive and negative emotional states in rats can be measured by monitoring their ultrasonic vocalizations (USVs); 50 kHz USVs align with hedonic emotional states while 22 kHz USVs align with aversive emotional states [1]. Frequency modulated 50 kHz calls (hedonic calls) are elicited by a wide variety of hedonic/rewarding stimuli, with positive social interactions (e.g., play) being the most effective elicitor of these calls [2]. Likewise, the most robust elicitor of positive affect in humans is also positive social interactions [3, 4]. The functional neuroanatomy and pharmacology of these calls is consistent with what is known about positive affect in humans [2]. Conversely, 22 kHz USVs in rats and negative affect in humans is best elicited by aversive/submissive social interaction [1–4] and share a similar neuronal substrate [1]. Hedonic and aversive calls share the same common output pathway, the periaqueductal gray (PAG), which is essential for both vocalizations and emotional expression across all mammals [5, 6].

The following five core principles of emotional expression in rats [1] have been corroborated by recent human ethological studies [5–7]: [1] Active social interactions are the most robust elicitor of emotional responses; [2] The internal state of the organism, not the nature of the stimulus, determines the affective valence (i.e., positive vs negative); [3] Social interaction begins with positive affect, and negative affect builds up over time; [4] The switching point from approach to avoidance occurs when the ratio of hedonic to aversive calls reaches 4:1 (80% positive and 20% aversive); and [5] The culmination of these properties leads to the expression of emotion and the daily rhythm of mood, in which positive affect is associated with active wake behaviors and negative affect with passive wake behavior, which transitions to sleep.

Multiple studies show that active wake behavior is associated with positive affect, and that sleep deprivation and corresponding groggy wake is a robust elicitor of negative emotions [8–10]. The transition from energized wake (positive affect) to tiredness (negative affect) appears to coordinate the wake-to-sleep cycle, implying a circadian rhythm of emotion. This rhythm acts in concert with social activity [1, 9].

Cortical alpha oscillations appear to reflect the synaptic plasticity mechanisms that regulate the daily rhythm of arousal and mood. The daily rhythms of arousal/tiredness and positive/negative emotion are closely related to that of alpha/delta cortical EEG power [1, 3, 7, 10]. At a mechanistic level, alpha frequencies enhance synaptic strength as shown by an increase in long-term potentiation (LTP), whereas delta frequencies decrease synaptic strength as shown by an increase in long-term depotentiation (LTD) [11]. Enhanced plasticity during active wake and depotentiation during sleep is a well-established mechanism of memory formation and maintenance [1, 12].

At the molecular level, our team has found that the induction of hedonic calls is associated with an upregulation of AMPA- and NMDA-type glutamate receptors (NMDARs and AMPARs) and growth factors associated with enhanced synaptic plasticity [1]. These same receptors and growth factors are downregulated during aversive calls. Several researchers have reported that the circadian rhythm of sleep–wake follows a similar pattern of NMDAR and AMPAR expression, with active wake being associated with increased NMDAR and AMPAR expression and the converse for sleep [13, 14].

This article describes a series of experiments that were designed to test the hypothesis that alpha and delta rhythms, regulated by glutamatergic modulation in the MPFC-PAG circuit, play a key role in the downstream generation of emotional expression by vocalization via alterations in synaptic plasticity.

## Materials and methods

### Rat studies

#### Animals

Experiments were approved by Northwestern University (Evanston IL) or Northshore Hospital (Evanston IL) IACUC committees and carried out in accordance with the Guide for the *Care and Use of Laboratory Animals* as adopted and promulgated by the U.S. National Institutes of Health. Male 2- to 3-month-old Sprague–Dawley rats from Charles River (United States) were used (N = 159). Rats were housed in Lucite cages with aspen-wood chip bedding, maintained on a 12:12 light:dark cycle (lights on at 6:00 am), and given ad libitum access to Teklad lab chow (Envigo, United States) and tap water throughout the study.

#### Surgeries

Rats were anesthetized with isoflurane (5% induction and 2–3% maintenance; 15–20 min total duration) and implanted with skull screws to record cortical EEG (Pinnacle, USA). Intercranial medial prefrontal cortex (MPFC) and PAG recording (monopolar) and stimulating (bipolar) insulated stainless steel electrodes (0.2 mm, diameter; Plastics One, USA) were also implanted. Animals were given 7 days to recover before the start of testing. EEG signals were captured via a tethered system (A-M systems, USA). Data were acquired at 10 kHz using an A-M systems (USA) amplifier with high- (0.1 Hz) and low-pass (100 kHz) filters and digitized using Data Wave acquisition software (A-M systems, USA). Data were analyzed using Brain Products Analyzer 2 software (Germany).

#### Physical and electrical stimulation-induced ultrasonic vocalizations

Animals first received physical stimulation to induce calling [15]. Physical stimulation consisted of 15 s of the experimenter rapidly moving their fingers across the animal’s back with a focus on the neck. This was followed by the experimenter quickly turning the animal over on its back, tickling the ventral surface rapidly for a few [2–5] seconds, and then releasing the animal. Cycles of physical stimulation were followed by 15 s cycles of no stimulation.

Once animals reliably exhibited ultrasonic vocalizations during the no-stimulation period, rats received 30 s of bipolar electrical stimulation of the medial prefrontal cortex at either the theta/alpha frequency (6 Hz; 70 uS 1 mA bipolar stimulation with an inter-stimulus interval of 167 ms, with 5 pulse trains at 100 Hz) or delta frequency (2 Hz; inter stimulus interval of 500 ms, and 15 pulse trains at 100 Hz) in a random manner. Both alpha and delta trains consisted of 30 pulses/second, thus controlling the total amount of stimulation. Vocalizations were recorded and rates of hedonic and aversive USVs were quantified on spectrograms using blind methods (Avisoft, Germany).

This manuscript reports on rat-to-human translational studies. The definition of alpha frequency is different between the species. The dominant frequency in the rat EEG as measured by power spectral density is 6–7 Hz, whereas the dominant EEG frequency in humans is 10 Hz [16], which is referred to as alpha [17]. We define the 6–7 Hz range in the rat as alpha; however, 6–7 Hz is typically referred to as theta in rodent literature [18]. To clarify this, we frequently use “theta/alpha” when referring to rats.

#### Closed-loop stimulation studies

EEG recordings from MPFC skull electrodes were collected from freely behaving rats. Real time alpha-delta ratios were calculated every 30 s using a custom data wave script (A-M Systems, USA). The threshold for closed-loop stimulation was set for each animal based on the mean and standard deviation alpha delta ratio during the pre-stimulation baseline for each animal (3 × SD of baseline alpha-delta ratio). Theta/alpha train stimulation (as described above) was delivered either once the threshold was reached (experimental) or at a yoked random control timepoint (control). Aversive USVs were induced either by moderate repeated pinching stimulation at the nape of the rat’s neck [19] across a 3 min test session, or 23 h of sleep deprivation by gentle handing (Pinnacle, USA; [9]). For the tactile stimulation group, 5 s of theta/alpha train stimulation was given, with a 30 s time out. For the sleep deprivation group, 30 s of theta/alpha train stimulation was given with a 15 min time out.

#### Operant self-administration/avoidance of hedonic or aversive tactile stimulation for quantitative electroencephalogram (qEEG) measures of affective states

Animals were placed singly into a 45 × 30 × 20 cm box and were given 3 s to produce an operant response (nose poke). The operant response immediately produced experimenter-delivered tactile stimulation to the animals. At the end of the 3 s, tactile stimulation was delivered regardless of the operant response behavior; these are the 3 s timeouts in each 4 min period. During the first 4 min, animals received 3 s of hedonic tickling, followed by 3 s of timeout. During the second 4 min, 3 s of neutral light touch was administered with the same 3 s timeout. In the final 4 min, 3 s of aversive tactile stimulation (moderate pinching at the nape of the neck) was administered with the same 3 s timeout. Operant responses, ultrasonic calls, and qEEG were quantified during each 4 min period, and motor-evoked potentials that were time-locked to the onset of USVs were also quantified.

#### Multielectrode array (MEA) recordings in vitro

Primary cortical cultures (E18 rat or P0 mouse) were plated onto 48-well MEA plates (Axion Biosystems, USA). Multi-unit action potential rates were determined by filtering the data using an IIR filter (low cutoff 300 Hz; high cutoff 3000 Hz) and applying a 4 × RMS threshold (Neuroexplorer, USA). To determine the dose of JB2 (IGF2R positive modulator, 20), 21 days-*in-vitro* (DIV) primary cortical neurons were analyzed 1 h after JB2 administration for field potential recordings by 48-well MEAs (Maestro, Axion biosystems). Stimulating a central electrode (100 µA, 100 µs) of each array produced single shock-induced field potentials, and potentials were recorded from the remaining 15 electrodes per well. The initial field potential slope after the stimulation artifact was used for quantification. In our preparation, we found that > 90% of this initial field potential slope was NBQX (AMPAR antagonist) sensitive [20]. NMDAR dependence was also tested with CPP (10 uM). To measure the mean firing rate, electrodes with a response of more than 0.5 Hz were considered for the analysis. Excitatory and inhibitory neurons were differentiated by means of interevent histograms between the various units/electrodes in each well (Neuroexplorer, USA). The firing of a target neuron that is associated with an immediate suppression of the firing of neighboring neurons is classified as inhibitory. The remaining neurons are classified as excitatory, with target firing leading to no change or an increase in firing rates of neighboring neurons.

#### In vivo studies of excitatory synaptic strength (fEPSPS)

Field potentials from freely behaving tethered rats were induced using a bipolar electrode that delivered a single shock (10 µA, 70 µs) every 30 s in layer 2–3 of the rat MPFC. Results were recorded in layer 4, and the initial slope after the stimulation artifact was quantified. Importantly, only electrode stimulation and recording pairs in which the field potential recordings were significantly attenuated by the AMPAR antagonist NBQX (10 mg/kg, IP; Sigma, USA) were used for analysis. The NMDAR antagonist CPP (10 mg/kg IP; Sigma, USA), and the IGF2R activator JB2 (1 mg/kg SC; Creative biomart, USA) were also used. Synaptic potentiation (LTP) was induced using three 30 s theta trains (three 70 µs stimulations with an inter-stimulation interval of 10 ms, and an inter-train interval of 167 ms) with 30 s of no stimulation between stimulation blocks. Depotentiation (LTD) was induced by three 30 s delta trains (three 70 µs stimulations with an inter-stimulation interval of 10 ms, and an inter-train interval of 500 ms).

#### Self-stimulation studies

Rats were placed singly in an arena (52 × 52 × 32 cm) with three novel objects located in the center. The objects were randomly assigned to one of three conditions: 1 s of theta train stimulation (three 70 µs stimulations with an inter-stimulation interval of 10 ms, and an inter-train interval of 167 ms); 1 s of delta train stimulation (nine 70 µs stimulations with an inter-stimulation interval of 10 ms, and an inter-train interval of 500 ms); or sham stimulation. Animals were allowed to explore the objects for 5 min, and nose contact with the object was used as the operant response. These responses were recorded in a blinded manner. Each operant response was followed by a 4 s time-out. Given the results of the previous study, the hypothesis was that rats should show greater levels of operant self-administration of theta/alpha frequency electrical stimulation as compared to delta frequency electrical stimulation, and that non-reinforced stimulation should lead to intermediate operant responding.

#### Clinical dataset mining

A public domain EEG dataset was used to evaluate emotional expression in humans [21]. Subjects (n = 31) were asked to imagine separately joy and sadness and instructed to press a button once they felt the requested emotion. Data was collected using scalp EEGs. Data for each emotional state was analyzed by comparing the 30 s after the onset of emotion to the 30 s of baseline EEG that preceded the button press. Power spectral density plots were generated using normalized power (1.5–30 Hz). A region of interest (ROI) was chosen based on contiguous or near contiguous frontal electrodes that showed opposing alpha and delta EEG responses to joy and sadness and that corresponded to the placement of the rat electrodes. These electrodes were E1-2, F5-9, F15-17, E31-32 (256 electrode Montague Biosemi, Netherlands).

#### Statistical analyses

Prism (GraphPad, USA) was used for all statistical analyses. Normality was first confirmed by the D'Agostino-Pearson test, followed by ANOVA followed by a Dunnetts post hoc test for multiple comparisons. For non-gaussian data, a Mann–Whitney U test was performed.

## Results

### Identifying a fingerprint for emotional valance in rats

**Fig. 1:**
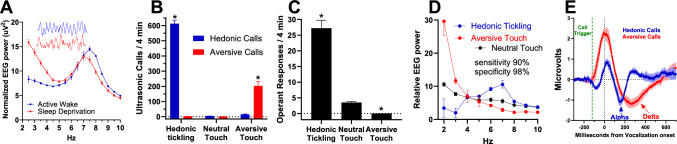
Identification of an EEG fingerprint of emotional valance in rats. **A** Medial prefrontal cortex qEEG from freely behaving isolate-housed animals during the first 1 h of lights off (active wake; blue line) and after 23 h of sleep deprivation (groggy wake; red line); Inset-representative 2 s EEG traces with a y axis range of 3 uV per trace. **B** Hedonic tickling shows increased hedonic USVs, whereas aversive tactile stimulation shows increased aversive calls. **C** Hedonic tickling is rewarding, and aversive tactile stimulation is avoided, as measured by operant self-administration. **D** Medial prefrontal cortex qEEG during hedonic and aversive tactile stimulation resembles an active wake and groggy wake pattern, respectively, (inset) machine learning can discriminate hedonic from aversive EEG with high sensitivity and specificity using a convolutional neuronal network. **E** EEG in the PAG is time locked to hedonic or aversive ultrasonic vocalization onset. The PAG is the final common output pathway for vocalizations, and the motor-evoked potential is time locked to vocalization onset, generating a single alpha wave for hedonic calls and a single delta wave for aversive calls, which also corresponds the average call duration of each call type

As shown in Fig. 1A, active wake behavior during the first 1 h of lights off (the beginning of the active phase for rats) is associated with the highest levels of hedonic USVs [9], and qEEG measures show an elevated alpha-delta ratio as compared to the prolonged sleep deprivation (t (10) = 9.835, P < 0.05). Figure 1B shows that hedonic tickling is associated with increased rates of hedonic calls (t (4) = 27.2, P < 0.05), and aversive stimulation is associated with increased rates of aversive calls (t (4) = 6.8, P < 0.05), whereas neutral touch does not change either hedonic or aversive calls (t (4) = 1.8 P > 0.05). As Fig. 1C shows, stimulation that induces hedonic calls increased operant responding for self-administration when compared to the effects of neutral touch (t (6) = 9.45, P < 0.05), and aversive stimulation showed fewer (zero) operant responses as compared to non-reinforced operant responses (Mann–Whitney U = 0, P < 0.05). Figure 1D presents qEEG results from cortical electrodes that show elevated alpha-delta ratios in hedonic tickling as compared to aversive tactile stimulation (F (2, 21) = 13.21, P < 0.05; P < 0.05 Dunnet’s post hoc test hedonic vs neutral and aversive vs neutral). Figure 1E shows results of PAG recording electrodes that show alpha and delta waves that were time-locked to the generation of hedonic and aversive calls. Notably, the median duration of hedonic and aversive calls approximates the duration of single alpha and delta waves, respectively [1].

### Externally applied alpha and delta frequency electrical stimulation to the MPFC induces positive and negative affect respectively, and closed-loop alpha frequency stimulation can prevent negative affect

**Fig. 2:**
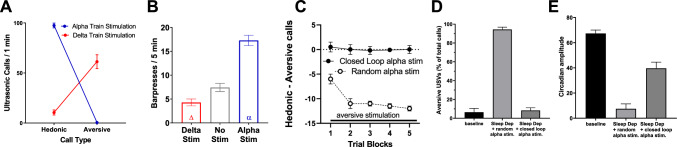
Medial prefrontal cortex alpha and delta frequency electrical stimulation induces positive and negative affect, respectively, and closed-loop alpha frequency stimulation can prevent negative affect. **A**, **B** MPFC electrical stimulation of the brain using alpha frequency stimulation is associated with increased hedonic calls and operant self-stimulation rates, while increased delta frequency stimulation is associated with increased aversive calls and reduced self-stimulation rates. **C**–**E** Alpha frequency electrical stimulation delivered during the start of delta EEG (closed loop) prevents: (**C**) the transition from positive to negative affect during aversive tickling, prevents (**D**) aversive vocalizations, and partially restores (**E**) circadian rhythms during sleep deprivation

As shown in Fig. 2A, theta/alpha frequency electrical brain stimulation (vs. yoked random stimulation) of the MPFC increases the number of hedonic calls. Delta frequency electrical stimulation increases the number of aversive calls. Both were indexed by a stimulation X call interaction (F (1, 20) = 360.3, P < 0.05) As shown in Fig. 2B, similar effects were seen with operant self-stimulation rates (F (2, 39) = 56.69, P < 0.05; P < 0.05 Dunnet’s post hoc test delta stim vs. neutral, and alpha stim vs neutral). Closed-loop theta/alpha frequency electrical stimulation prevents the induction of aversive USVs in response to aversive tactile stimulation, as can be seen in Fig. 2C. (F (1, 30) = 443.3, P < 0.05; P < 0.05), Fisher’s PLSD post hoc test comparing closed-loop vs random test at each trail block). Closed-loop alpha frequency electrical stimulation in sleep deprived rats (Fig. 2D) reduced aversive calls and (Fig. 2E) disruptions in circadian rhythm amplitude along with partially restoring the sleep wake cycle in sleep deprivation (F (2, 12) = 248.5, 58.63, P < 0.05; P < 0.05, Dunnet’s post hoc test baseline vs. random alpha stim and closed-loop alpha stim vs random alpha stim).

### Active wake qEEG is associated with synaptic strengthening, while groggy wake qEEG is associated with synaptic weakening

**Fig. 3:**
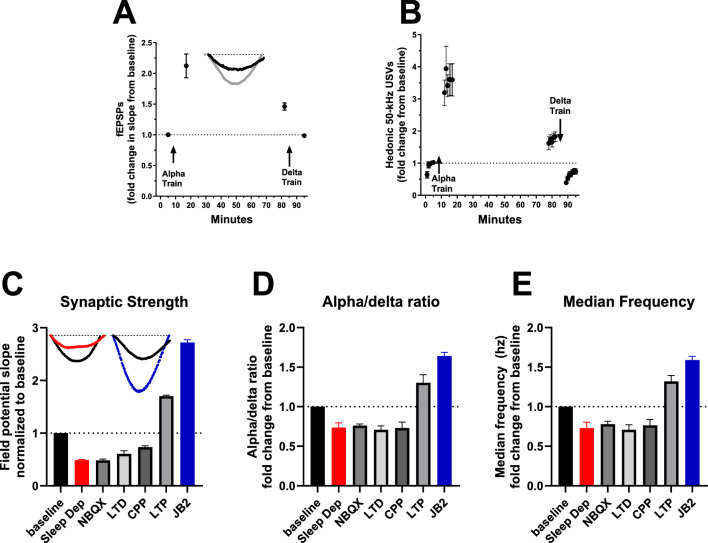
Active wake qEEG is associated with synaptic strengthening and groggy wake qEEG is associated with synaptic weakening. **A**, **B** MPFC alpha frequency electrical stimulation induces LTP and increases hedonic calling compared to a normalized baseline; both are reduced upon delta frequency electrical stimulation as compared to the long-term potentiated response. **C**–**E** MPFC synaptic strength (**C**) and active wake qEEG (**D**, **E**) are increased, as compared to a normalized baseline, by the induction of tetanus induced LTP (LTP) or growth factor induced LTP (JB2; 1 mg/kg SC), and inhibited, as compared to a normalized baseline, by sleep deprivation, NMDAR antagonism (CPP; 10 mg/kg IP) or AMPAR antagonism (NBQX; 10 mg/kg IP). Insets- representative excitatory field potential recordings from a single rats 1 h after tetanus / LTP (grey), after receiving 23 h of sleep deprivation (red), JB2 (1 mg/kg SC, Blue), as compared to baseline (black). Insets, Dotted lines represent 0 microvolt, and the peak of the JB2 field is at − 5.7 microvolts; Four to 14 ms post-stimulation is the x axis time range, and all traces are on the same scales

As shown in Fig. 3A, B, MPFC electrical brain stimulation induced enhancement changes in synaptic strength and 50 kHz USVs; both show a long-term potentiation following theta/alpha frequency electrical stimulation and short-term depotentiation following delta train stimulation (field potentials t (12) = 7.89, 7.41, P < 0.05; hedonic calls t (12) = 7.03, 9.86, P < 0.05). Synaptic strength (Fig. 3C), alpha-delta ratio (Fig. 3D), and median power spectral density (PSD) frequency (Fig. 3E), all decreased in home cage recordings following 23 h of sleep deprivation, 0.5–1 h after administration of the AMPA receptor antagonist NBQX (10 mg/kg IP), 1 h after the induction of LTD, and 0.5–1 h after administration of the NMDA receptor antagonist CPP (10 mg/kg IP; [22]); Conversely, 24 h after administration of the IGF2R growth factor positive modulator JB2 (1 mg/kg SC; [20]) or theta-train stimulation of LTP, each of these measures was increased [(F 6, 29) = 713.5, 25.8, 27.49, P < 0.05; Dunnet’s test baseline vs each dosing group separately].

### At the single neuron level, synaptic strength and EEG speed is positively correlated to the firing rate of excitatory, but not inhibitory, neurons

**Fig. 4:**
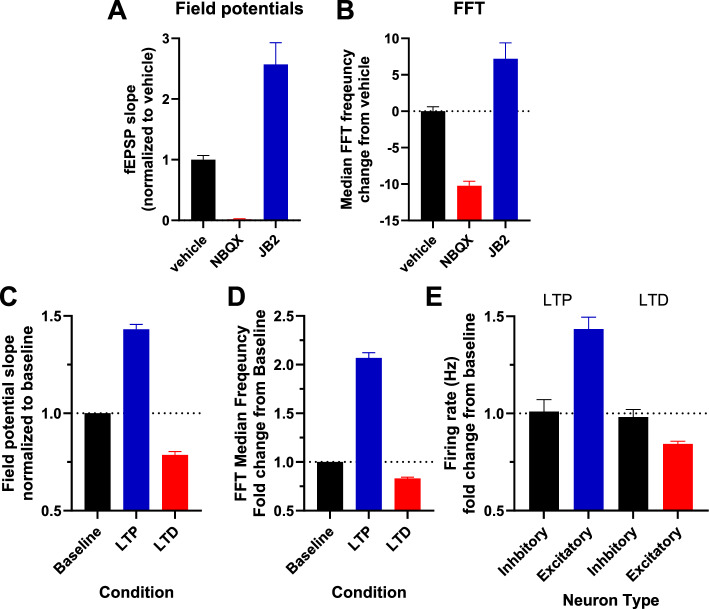
At the single neuron level, synaptic strength and FFT speed is positively correlated to the firing rate of excitatory, but not inhibitory, neurons. The electrophysiological properties of primary rat cortical cultures (21 DIV) were measured in vitro by MEA. **A** JB2 (1 uM) enhanced and NBQX (1 uM) inhibited synaptic strength, **B** and JB2 increased and NBQX decreased the median speed of the FFT transformed MEA recordings measured in Hz. **C**, **D** Similar effects were seen as shown in A-B with LTP and LTD induction using theta and delta trains respectively. **E** The firing rate of excitatory neurons increased after LTP, but decreased after LTD

Figure 4 shows that both field potentials (A) and median frequency of the power spectral density plots (B) in MEA recordings from rat cortical neuronal cultures are reduced by NBQX (1 uM), and increased by JB2 (1 uM) 1 h after administration (F (2, 686) = 171.6, P < 0.05; P < 0.05 Dunnet’s test vehicle vs. all other groups separately: F (2, 1137) = 73.70, P < 0.05; P < 0.05 Dunnet’s test vehicle vs. all other groups separately). Similar increases were seen 1 h after LTP induction with theta train stimulation and decreases 1 h after LTD induction with delta frequency electrical stimulation, as shown in C and D (F (2, 1556) = 299.4, P < 0.05; P < 0.05 Dunnet’s test vehicle vs. all other groups separately: F (2, 964) = 464.7, P < 0.05; P < 0.05 Dunnet’s test vehicle vs. all other groups separately). Figure 4E shows the firing rate of neurons after LTP and LTD induction. Only excitatory neurons were potentiated/de-potentiated by LTP/LTD (t (106) = 2.83 P < 0.05; t (208) = 2.60 P < 0.05 comparing excitatory to inhibitory neurons).

### Translation of the EEG fingerprint of emotional valance in humans

**Fig. 5:**
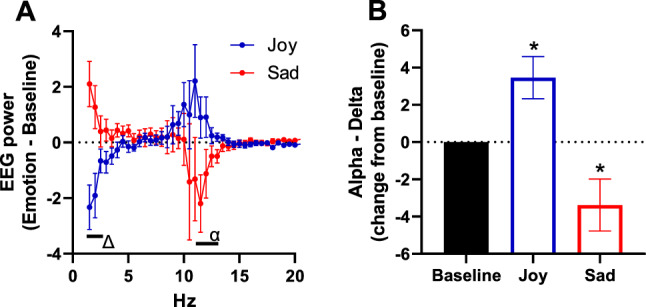
Translation of the EEG fingerprint of emotional valance in humans. Normal volunteers were asked to imagine joy and sadness during scalp EEG recording and instructed to indicate with a button press when the feeling state was successfully induced. qEEG during the 30 s after the emotional onset was compared to the 30 s of baseline before emotional induction. A contiguous region of interest was identified comprising the frontal central scalp electrodes and extending into the left frontal electrodes, and FFT data was averaged across these electrodes. **A** In a similar manner as seen in rats, sadness increased delta power and decreased alpha power and subsequently slowed overall qEEG, whereas joy produced the opposite responses. **B** Alpha power was increased relative to delta power during joy and decreased during sadness. * P < .05 within subjects 2-tailed t-test compared to the respective baseline

**Fig. 6:**
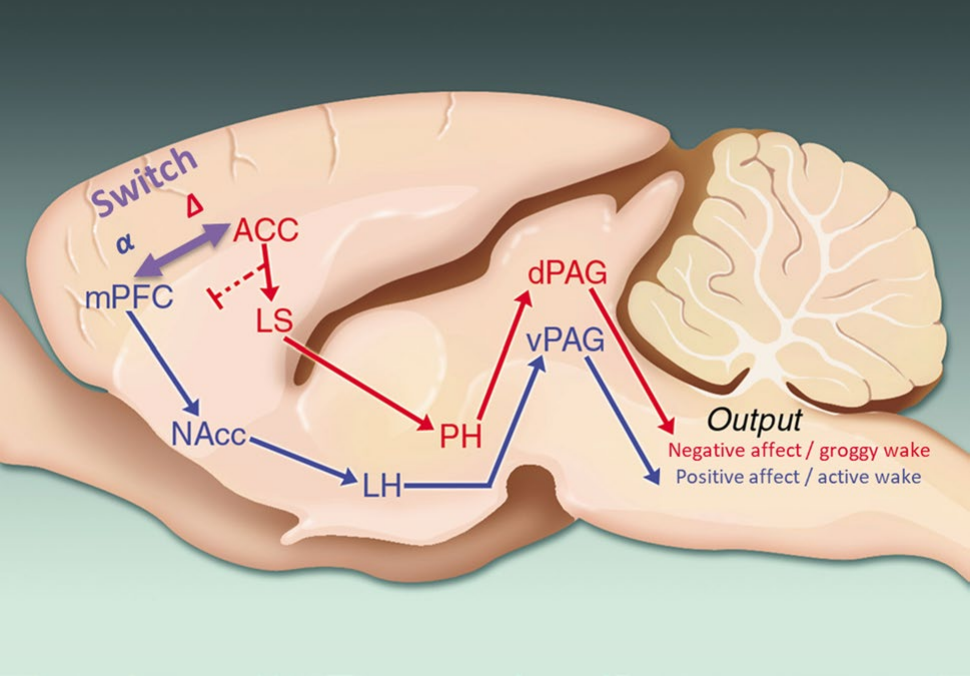
Emotional valance switch model. During active wake behavior, MPFC alpha power predominates. In concert with social stimuli, MPFC alpha power activates the hedonic ultrasonic vocalization/reward circuit, and this switch is driven by an LTP/LTD mechanism involving NMDA and growth factor activation. In humans, a similar switch regulates positive and negative mood

Figure 5 shows that similar patterns were observed in humans as those in rats: increased alpha and decreased delta power were seen with positive affect (joy), and opposite effects were seen in negative affect (sadness) (F (2, 88) = 11.11, P < 0.05; P < 0.05 Dunnet’s test joy vs neutral and sad vs neutral). A positive affect correlated with a faster EEG, and a negative affect correlated with a slower EEG, as measured by the slope of the change score from 2 to 10 Hz (data not shown). There were no sex effects seen in these measures (all P’s > 0.05). The proposed neuronal circuit for the generation of positive and negative affect, along with the MPFC alpha delta switch, is shown in Fig. 6.

## Discussion

The results of the present studies show that positive affect is associated with synaptic plasticity and active wake EEG patterns. Conversely, negative affect is related to decreased synaptic plasticity and groggy wake EEG. An NMDAR and growth-factor-receptor-dependent LTP mechanism in the MPFC drives positive affect, whereas reducing NMDAR and growth factor receptor activity leads to an increase in LTD in the MPFC, driving the switch to negative affect.

An NMDAR and growth-factor-receptor-dependent LTP mechanism in the MPFC-PAG circuit appears to regulate positive emotions via the synchronization of the alpha rhythm. The neuronal circuit associated with the ability to express emotions has been well described [1, 5, 6]: The MPFC is the flexible/adaptable node of the circuit, whereas the PAG, pons, and medulla components of the circuit drive the behavior output. That is, the MPFC is the decision-making part of the circuit, capable of controlling the switch between positive and negative affect, and appears to be responsible for modifying or changing the emotional state. This leads to the hypothesis that synchronized alpha in the MPFC entrains the PAG to the alpha rhythm, which leads to production of hedonic ultrasonic vocalizations, and sniffing exploration behavior. These behaviors have been shown to occur at the alpha frequency (~ 6 times per second; 1). Furthermore, we suggest that the synchrony of alpha oscillations in the MPFC is due to enhanced Hebbian synaptic plasticity in excitatory neurons with similar pharmacology (NMDAR, AMPAR, growth factors) as is seen with canonical LTP [11].

Negative emotional states occur when the MPFC-PAG circuit switches from alpha to delta synchronization. This suggests that the delta rhythm is related to decreased excitatory activity and a net decrease in excitatory-inhibitory balance. It also supports the idea that the synchrony of delta oscillations in the MPFC is due to an enhanced LTD process, which is associated with a downregulation of NMDAR, AMPAR, growth factor activity [11].

We have identified a novel mechanism, the alpha-delta switch, that is responsible for modifying or generating emotional state and how the brain can switch between positive and negative affect. The levels of alpha and delta power also play a part in the generation of synaptic plasticity which in turn is regulated by the glutamatergic neurotransmitter system. Measuring the alpha-delta ratio should help with the development of novel glutamatergic-based therapeutics for a variety of psychiatric disorders.

## Data Availability

The datasets generated during and/or analyzed during the current study are available from the corresponding author on reasonable request.

## Abbreviations

ACC: Anterior cingulate cortex
AMPARs: α-Amino-3-hydroxy-5-methyl-4-isoxazolepropionic acid-type glutamate receptors
CPP: ( ±)-3-(2-Carboxypiperazin-4-yl)propyl-1-phosphonic acid
DIV: Days-in-vitro
dPAG: Dorsal periaqueductal gray
fEPSPS: Field excitatory postsynaptic potentials
LH: Lateral hypothalamus
LS: Lateral septum
LTD: Long-term depotentiation
LTP: Long-term potentiation
MEAs: Multielectrode array
MPFC: Medial prefrontal cortex
NAcc: Nucleus accumbens
NBQX: 1,2,3,4-Tetrahydro-6-nitro-2,3-dioxo-benzo[f]quinoxaline-7-sulfonamide hydrate
NMDARs: N-methyl-d-aspartate -type glutamate receptors
PAG: Periaqueductal gray
PH: Posterior hypothalamus
PSD: Power spectral density
qEEG: Quantitative electroencephalograms
ROI: Region of interest
SD: Sprague–Dawley
USVs: Ultrasonic vocalizations
vPAG: Ventral periaqueductal gray
